# Supplementary material for: A Photoluminescent Colorimetric Probe of Bovine Serum Albumin-Stabilized Gold Nanoclusters for New Psychoactive Substances: Cathinone Drugs in Seized Street Samples
Source: Sensors (Basel). 2019 Aug 15;19(16):3554. doi: 10.3390/s19163554 (PMC6721205; doi:10.3390/s19163554)
Supplement: Supplementary file 1 [file sensors-19-03554-s001.pdf]

Supporting Information

A Photoluminescent Colorimetric Probe of Bovine Serum Albumin-Stabilized Gold Nanoclusters for New Psychoactive Substances: Cathinone-Drugs in Seized Street Samples

Yao-Te Yen[1,2,*], Ting-Yueh Chen[1], Chun-Yu Chen[3], Chi-Lun Chang[1], San-Chong Chyueh[1] and Huan-Tsung Chang[2]

[1] Department of Forensic Science, Investigation Bureau, Ministry of Justice, Xindian District, New Taipei City 23149, Taiwan

[2] Department of Chemistry, National Taiwan University, Taipei 10617, Taiwan

[3] Department of Chemistry, National Cheng-Kung University, Tainan 70101, Taiwan

Correspondence: Yao-Te Yen, Department of Forensic Science, Investigation Bureau, Ministry of Justice, Xindian District, New Taipei City 23149, Taiwan; E-mail: d05223113@ntu.edu.tw; Tel: 011-886-2-29112241-3714

Supporting Information I

Sodium borohydride (8 mg) was added to 2 mL of an aqueous solution containing 4-chloromethcathinone (15 mg) in a glass tube. After a reaction time of 1 h, the cruel was adjusted to be alkaline by adding 0.5 mL of 0.1 M $NaOH_{(aq)}$; next, the desired compound was extracted three times using a mixture (2 mL) of ethyl acetate and dichloromethane (v:v = 1:1). The organic layer was evaporated to dryness under a gentle stream of nitrogen at 40°C, and the obtained compound was identified in D_2O using ^1H nuclear magnetic resonance spectroscopy (as shown in Figure S1). The exact mass of obtained compound $[M+H]^+$ is 200.08 amu measured by using Liquid Chromatography–Orbitrap Spectrometry (Q Exactive Plus, Thermo Scientific, Bremen, Germany)

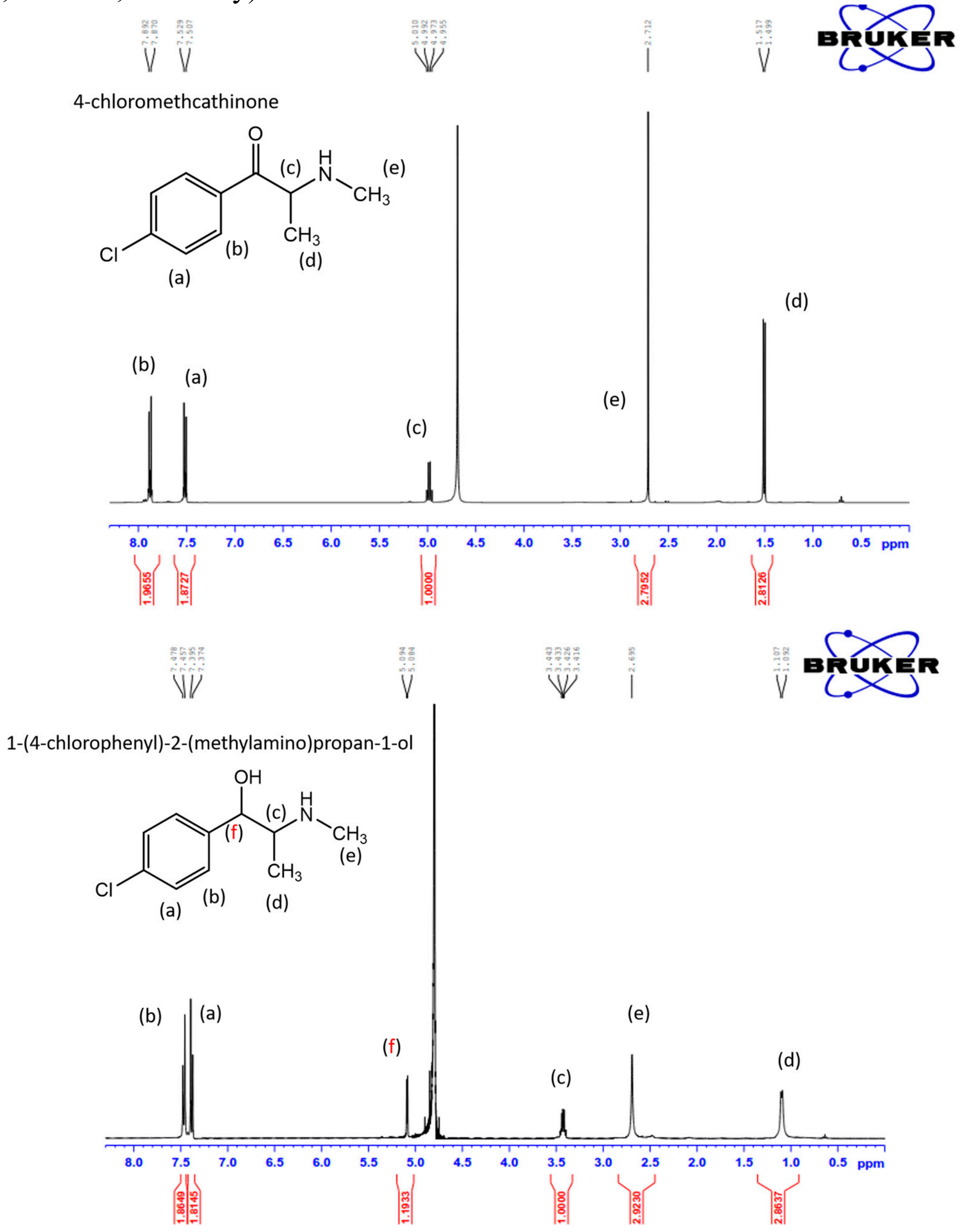

Figure S1. NMR spectrum of 4-chloromethcathinone and 1-(4-chlorophenyl)-2-(methylamino)propan-1-ol.

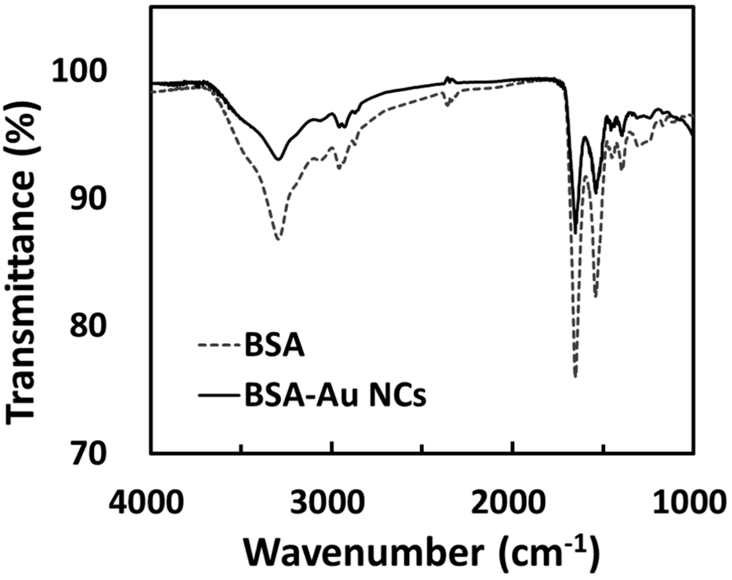

Figure S2. FTIR spectrum of BSA-Au NCs and BSA.

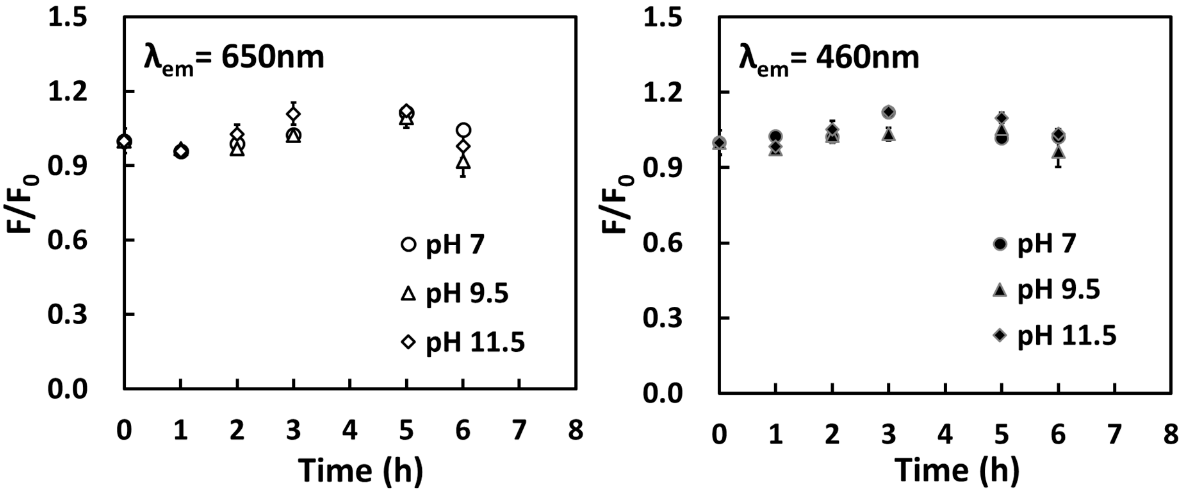

Figure S3. Stability of BSA-Au NCs with different pH values over time. (A) Emission at 650 nm, and (B) emission at 460 nm of BSA-Au NCs probe solution. F_0 and F are the PL intensities of BSA-Au NCs at 650 nm or 460 nm at the beginning and at the different time, respectively

Table S1. Structures of cathinone-drugs and other tested drugs.

Name	Structure
4-Chloromethcathinone	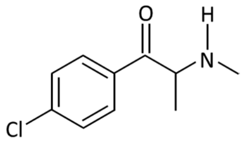
3-Methoxymethcathinone	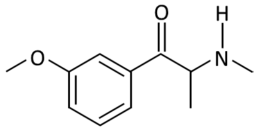
Mexedrone	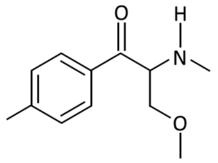
4-Methyl-α-ethylaminopentiophenone (4-MEAPP)	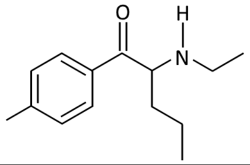
4'-chloro-α-Pyrrolidinopropiophenone (4'-chloro-PPP)	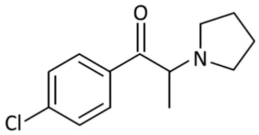
Butylone	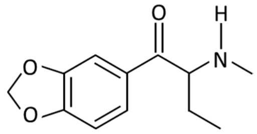
Pentylone	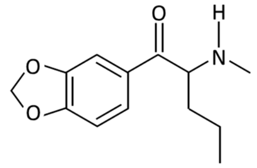
Ethylone	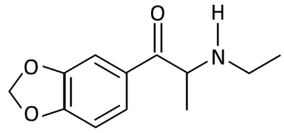
Dibutylone	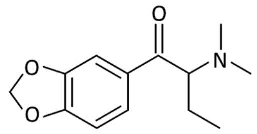
N-ethyl Pentylone	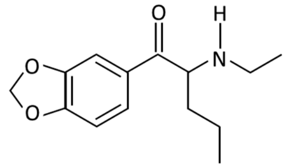

Name	Structure
Amphetamine	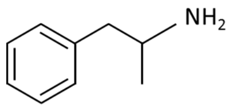
Ketamine	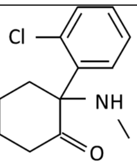
Methoxetamine	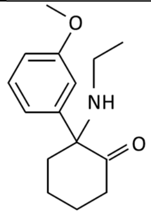
Cocaine	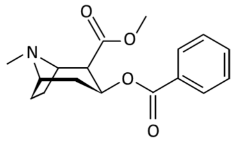
2,5-dimethoxy-4-bromophenethylamine (2C-B)	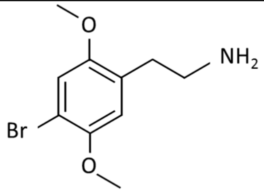
3,4-methylenedioxymethamphetamine (MDMA)	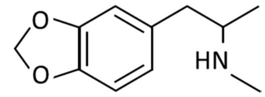
ACHMINACA	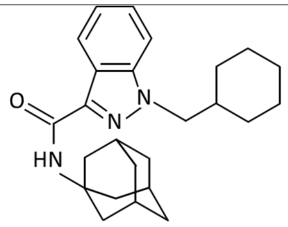
5-Chloro- AKB48	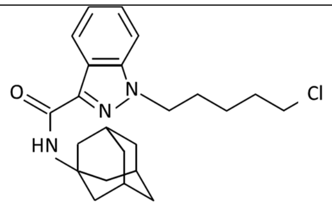

Name	Structure
Fentanyl	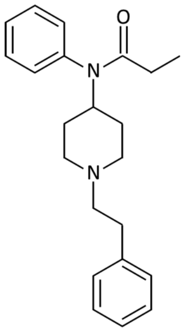
Etizolam	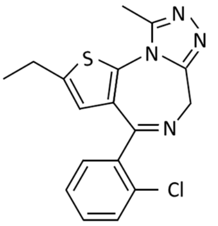
4-chloro-N,N-DMC	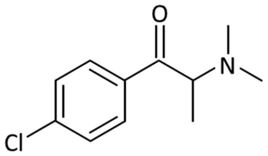
Heroin	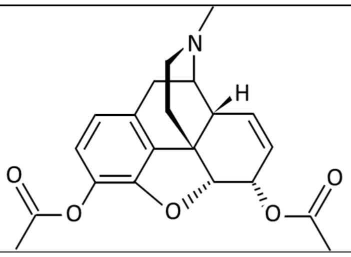
